# PHACE syndrome: report of five cases^؆^

**DOI:** 10.1016/j.abd.2026.501310

**Published:** 2026-03-25

**Authors:** Julia Maria de Oliveira Neumayer, Ana Clara Maia Palhano, Milene Tiburcio Narenti Ferradoza, Luciana Paula Samorano, Maria Cecília Rivitti-Machado, Zilda Najjar Prado de Oliveira

**Affiliations:** Department of Pediatric Dermatology, Faculty of Medicine, Hospital das Clínicas, Universidade de São Paulo, São Paulo, SP, Brazil

Dear Editor,

Infantile Hemangioma (IH) is the most frequent benign tumor in the pediatric population, occurring in up to 10% of children less than 1-year and usually involving the head and neck region, with female predominance.^1^, ^2^ PHACE syndrome (Posterior fossa malformations, Hemangiomas, Arterial anomalies, Coarctation of the aorta/Cardiac defects, and Eye abnormalities) is a rare neurocutaneous syndrome characterized by the association of infantile hemangioma larger than 5 cm on the face, scalp or cervical region and structural anomalies of the brain, cerebral vasculature, eyes, sternum, heart, and/or aorta.^1^, ^3^ A modified acronym, PHACES, has been suggested to include Sternal abnormalities.^1^, ^3^ The pathogenesis of PHACE syndrome remains unknown, and there is no evidence of genetic abnormalities contributing to its development. PHACE syndrome also seems to have female predominance, and it occurs in 2%‒3% of all IH cases and 20% of patients with large or segmental facial hemangiomas.^1^, ^4^ Five patients with PHACE syndrome were followed at the Pediatric Dermatology Outpatient Clinic of Hospital das Clínicas, Faculdade de Medicina, Universidade de São Paulo, for an average follow-up period of five years. All the patients were female and had a large facial infantile hemangioma that developed at birth or soon thereafter. Prematurity was observed in one patient. Family history of PHACE syndrome or parents’ consanguinity was not observed. Four of five patients presented with brain and cardiac anomalies. Ocular and sternal abnormality was identified in two and one patient, respectively. All patients were treated with oral and/or topical beta-blockers. Patients' features and clinical findings are described in Table 1. Magnetic Resonance Imaging (MRI) and Magnetic Resonance Angiography (MRA) findings are presented in Figs. 1 and 2A, respectively. Fig. 2B illustrates sternal aplasia and thoracic cutis aplasia. The evolution of the lesions after oral propranolol treatment is shown in Fig. 3.

**Table 1:** Features and clinical findings of patients with PHACE syndrome.

	Case 1	Case 2	Case 3	Case 4	Case 5
Prematurity	No	Yes	No	No	No
Time of follow-up	32 months	18 months	87 months	86 months	89 months
Age at the beginning of follow-up	4 months and 29 days	2 years and 4 months	1 year	1 year and 10 months	1 month and 15 days
HI topography	Right periocular	Left frontotemporal/ Frontonasal	Right temple, eyelid and pinna hemangioma	Right hemiface and retroauricular	Frontonasal
Brain anomalies	Right hemisphere cerebellar dysplasia and anterior cerebral artery A1 segment hypoplasia	Seizure	Tortuosity, dysplasia and anomalous origin of some cerebral arteries (right internal carotid and intracranial arteries)	No	Disencephaly
Cardiac anomalies	No	Coarctation of the aorta and interatrial communication (IAC)	Interventricular communication (IVC)	Interventricular communication (IVC), patent arterial duct (PAD) and coarctation of the aorta (CoA)	Interatrial communication (IAC)
Ocular anomalies	Strabismus – right eye	Strabismus, right-side amaurosis and Coats disease	No	No	No
		Retinoblastoma			
Sternal anomalies	No	No	Sternal aplasia and thoracic cutis aplasia	No	No
Other anomalies	Speech delay	Left-sided vestibular schwannoma	No	No	Salmon patch
	Behavior disorder				
Treatment	Oral propranolol	Oral propranolol	Topical timolol	Oral propranolol	Oral propranolol
	Oral corticosteroids	Topical timolol		Topical timolol	Topical timolol
Treatment duration	Propranolol: 15 months	Propranolol: 18 months	28 months	Propranolol: 17 months	Propranolol (3 intervals of use)
				Timolol: 28 months	
	Oral corticosteroids: unknown	Timolol: 22 months			1^st^ Interval: 32 months
					2^nd^ Interval:7 months
					3^rd^ Interval: 15 months
					Total: 54 months
					Timolol: 52 months
Side-effects	No	No	No	Mild bronchospasm episodes	No
PHACE’s syndrome complications	No	No	No	No	No
IH treatment response	Effective	Effective	Effective	Effective	Recurrence

**Fig. 1:**
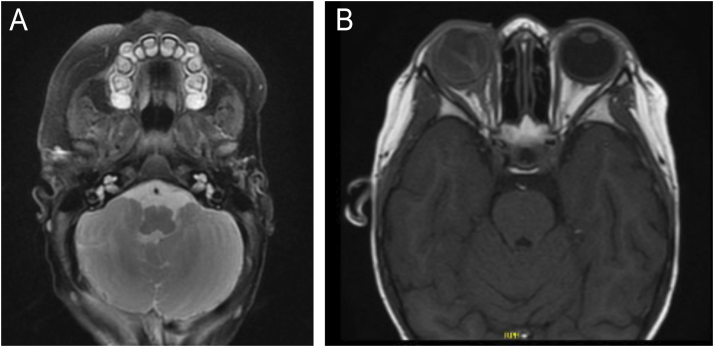
(A) MRI showing hypoplasia of the right cerebellum (case 1). (B) MRI showing right retinoblastoma (case 2).

**Fig. 2:**
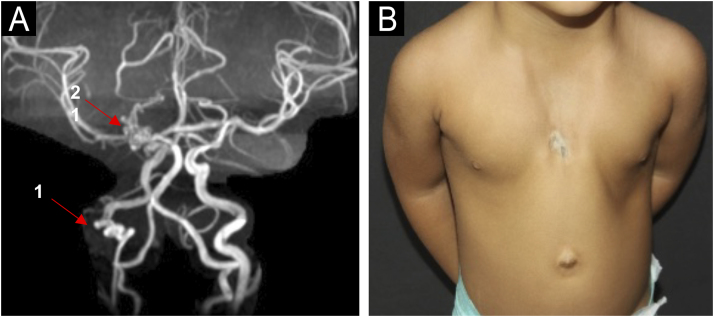
(A) MRA showing tortuous: 1) Right internal carotid artery and 2) right posterior cerebral artery and (B) Sternal aplasia and thoracic cutis aplasia (case 3).

**Fig. 3:**
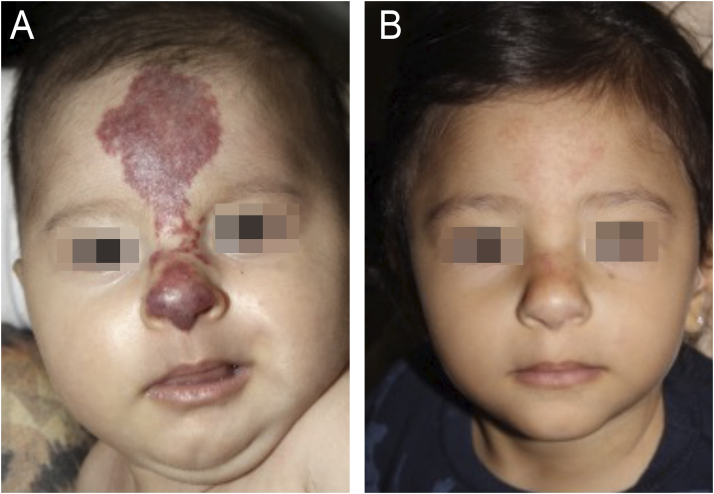
Female patient with right hemiface infantile hemangioma and PHACE syndrome (case 4). (A) At 2-years and 4-months of age after 17-months of treatment with oral propranolol (2 mg/kg/day). (B) At 7-years and 1-month of age, showing good response of the infantile hemangioma after 17-months of treatment with oral propranolol (2 mg/kg/day) and 2-years and 4-months of topical timolol.

The five cases described in this report illustrate the spectrum of features seen in PHACE syndrome, highlighting the heterogeneity of malformations associated with the condition.

The most common extracutaneous involvement is structural and vascular in the brain, followed by cardiac defects.^1^, ^3^, ^5^ The combined incidence of structural cerebral and cerebrovascular anomalies is greater than 50%.^2^ A variety of structural cardiac abnormalities have been reported in approximately one-third of PHACE patients.^2^ In our series, the majority of patients had brain and cardiac anomalies, as observed in the literature.^5^

Ocular anomalies are relatively rare in PHACE syndrome,^4^ however, we had 2 patients with ophthalmological abnormalities. One of these patients presented with a retinoblastoma, a rare malignant ocular tumor, which has been previously described in association with PHACE syndrome in two case reports.^4^, ^6^

One of our patients presented with a vestibular schwannoma. The occurrence of this tumor in PHACE syndrome patients is a rare finding, with scarce data in the literature.^7^

Speech delay is a common finding^1^, ^8^ and we identified in one of our patients.

Current literature suggests propranolol is effective in treating IH associated with PHACE syndrome.^3^, ^9^ Although the resolution of IH is well-documented during treatment with oral propranolol, its use in PHACE syndrome in patients who have cardiac and vascular malformations is controversial due to the risk of stroke.^1^, ^3^ Four of our five patients were treated with oral propranolol, and none experienced cerebrovascular complications. Careful examination and management by a multidisciplinary team are crucial to providing better care for these patients.^9^ The multidisciplinary group comprises neuroradiology, neurosurgery, neurology, cardiology, cardiothoracic surgery, dermatology, otorhinolaryngology, hematology-oncology, and plastic surgery.^1^ The initial physical examination of all suspected cases should include an assessment for ocular and sternal midline abnormalities, in addition to neurological, cardiac, and vascular evaluations.^1^ A screening echocardiogram should be executed and if abnormalities are identified, cardiac MRI and MRA are recommended.^1^, ^10^ Gadolinium MRI and MRA of the brain, neck, and aortic arch should also be implemented during follow-up for all suspected cases of PHACE syndrome. MRI is insufficient to detect all arterial abnormalities, requiring an MRA.^1^

We emphasize the importance of early recognition of the syndrome and evaluation of associated abnormalities, enabling effective interventions to prevent future complications. Further research into the long-term outcomes and better treatment strategies for this rare condition is necessary to improve both the quality of care and quality of life for affected children.

## ORCID ID

Ana Clara Maia Palhano: 0000-0002-0404-6482

Milene Tiburcio Narenti Ferradoza: 0000-0002-5864-7259

Luciana Paula Samorano: 0000-0001-7077-8553

Maria Cecília Rivitti-Machado: 0000-0003-2910-7330

Zilda Najjar Prado de Oliveira: 0000-0002-8596-1999

## Financial support

None declared.

## Authors' contributions

Julia Maria de Oliveira Neumayer: Study conception and planning; data collection, analysis and interpretation; preparation and writing of the manuscript; manuscript critical review; critical literature review; approval of the final version of the manuscript.

Ana Clara Maia Palhano: Data collection, analysis and interpretation; data and manuscript critical review; approval of the final version of the manuscript.

Milene Tiburcio Narenti Ferradoza: Data collection, analysis and interpretation; data and manuscript critical review; approval of the final version of the manuscript.

Luciana Paula Samorano: Intellectual participation in propaedeutic and therapeutic management of studied cases; data collection, analysis and interpretation; data and manuscript critical review; effective participation in research orientation; approval of the final version of the manuscript.

Maria Cecília Rivitti-Machado: Intellectual participation in propaedeutic and therapeutic management of studied cases; data collection, analysis and interpretation; data and manuscript critical review; effective participation in research orientation; approval of the final version of the manuscript.

Zilda Najjar Prado de Oliveira: Intellectual participation in propaedeutic and therapeutic management of studied cases; data collection, analysis and interpretation; data and manuscript critical review; effective participation in research orientation; approval of the final version of the manuscript.

## Research data availability

Does not apply.

## Conflicts of interest

None declared.
